# Editorial: Coronavirus Disease (COVID-19): The Impact and Role of Mass Media During the Pandemic

**DOI:** 10.3389/fpsyg.2021.729238

**Published:** 2021-08-23

**Authors:** Patrícia Arriaga, Francisco Esteves, Marina A. Pavlova, Nuno Piçarra

**Affiliations:** ^1^Department of Social and Organizational Psychology, Iscte-University Institute of Lisbon, CIS-IUL, Lisbon, Portugal; ^2^Department of Psychology and Social Work, Mid Sweden University, Östersund, Sweden; ^3^Department of Psychiatry and Psychotherapy, Medical School and University Hospital, Eberhard Karls University of Tübingen, Tübingen, Germany

**Keywords:** COVID-19, coronavirus disease, mass media, health communication, prevention, intervention, social behavioral changes

The outbreak of the coronavirus disease 2019 (COVID-19) has created a global health crisis that had a deep impact on the way we perceive our world and our everyday lives. Not only has the rate of contagion and patterns of transmission threatened our sense of agency, but the safety measures to contain the spread of the virus also required social and physical distancing, preventing us from finding solace in the company of others. Within this context, we launched our Research Topic on March 27th, 2020, and invited researchers to address *the Impact and Role of Mass Media During the Pandemic* on our lives at individual and social levels.

Despite all the hardships, disruption, and uncertainty brought by the pandemic, we received diverse and insightful manuscript proposals. Frontiers in Psychology published 15 articles, involving 61 authors from 8 countries, which were included in distinct specialized sections, including Health Psychology, Personality and Social Psychology, Emotion Science, and Organizational Psychology. Despite the diversity of this collective endeavor, the contributions fall into four areas of research: (1) the use of media in public health communication; (2) the diffusion of false information; (3) the compliance with the health recommendations; and (4) how media use relates to mental health and well-being.

A first line of research includes contributions examining the use of media in public health communication. Drawing on media messages used in previous health crises, such as Ebola and Zika, Hauer and Sood describe how health organizations use media. They offer a set of recommendations for COVID-19 related media messages, including the importance of message framing, interactive public forums with up-to-date information, and an honest communication about what is known and unknown about the pandemic and the virus. Following a content analysis approach, Parvin et al. studied the representations of COVID-19 in the opinion section of five Asian e-newspapers. The authors identified eight main issues (health and drugs, preparedness and awareness, social welfare and humanity, governance and institutions, the environment and wildlife, politics, innovation and technology, and the economy) and examined how e-newspapers from these countries attributed different weights to these issues and how this relates to the countries' cultural specificity. Raccanello et al. show how the internet can be a platform to disseminate a public campaign devised to inform adults about coping strategies that could help children and teenagers deal with the challenges of the pandemic. The authors examined the dissemination of the program through the analysis of website traffic, showing that in the 40 days following publication, the website reached 6,090 visits.

A second related line of research that drew the concern of researchers was the diffusion of false information about COVID-19 through the media. Lobato et al. examined the role of distinct individual differences (political orientation, social dominance orientation, traditionalism, conspiracy ideation, attitudes about science) on the willingness to share misinformation about COVID-19 over social media. The misinformation topics varied between the severity and spread of COVID-19, treatment and prevention, conspiracy theories, and miscellaneous unverifiable claims. Their results from 296 adult participants (Mage = 36.23; 117 women) suggest two different profiles. One indicating that those reporting more liberal positions and lower social dominance were less willing to share conspiracy misinformation. The other profile indicated that participants scoring high on social dominance and low in traditionalism were more willing to share both conspiracy and other miscellaneous claims, but less willing to share misinformation about the severity and spread of COVID-19. Their findings can have relevant contributions for the identification of specific individual profiles related to the widespread of distinct types of misinformation. Dhanani and Franz examined a sample of 1,141 adults (Mage = 44.66; 46.9% female, 74.7% White ethnic identity) living in the United States in March 2020. The authors examined how media consumption and information source were related to knowledge about COVID-19, the endorsement of misinformation about COVID-19, and prejudice toward Asian Americans. Higher levels of trust in informational sources such as public health organizations (e.g., Center for Disease Control) was associated with greater knowledge, lower endorsement of misinformation, and less prejudice toward Asian Americans. Media source was associated with distinct levels of knowledge, willingness to endorsement misinformation and prejudice toward American Asians, with social media use (e.g., Twitter, Facebook) being related with a lower knowledge about COVID-19, higher endorsement of misinformation, and stronger prejudice toward Asian Americans.

A third line of research addressed the factors that could contribute to compliance with the health recommendations to avoid the spread of the disease. Vai et al. studied early pre-lockdown risk perceptions about COVID-19 and the trust in media sources among 2,223 Italians (Mage = 36.4, 69.2% female). They found that the perceived usefulness of the containment measures (e.g., social distancing) was related to threat perception and efficacy beliefs. Lower threat perception was associated with less perception of utility of the containment measures. Although most participants considered themselves and others capable of taking preventive measures, they saw the measures as generally ineffective. Participants acknowledged using the internet as their main source of information and considered health organizations' websites as the most trustworthy source. Albeit frequently used, social media was in general considered an unreliable source of information. Tomczyk et al. studied knowledge about preventive behaviors, risk perception, stigmatizing attitudes (support for discrimination and blame), and sociodemographic data (e.g., age, gender, country of origin, education level, region, persons per household) as predictors of compliance with the behavioral recommendations among 157 Germans, (age range: 18–77 years, 80% female). Low compliance was associated with male gender, younger age, and lower public stigma. Regarding stigmatizing attitudes, the authors only found a relation between support for discrimination (i.e., support for compulsory measures) and higher intention to comply with recommendations. Mahmood et al. studied the relation between social media use, risk perception, preventive behaviors, and self-efficacy in a sample of 310 Pakistani adults (54.2% female). The authors found social media use to be positively related to self-efficacy and perceived threat, which were both positively related to preventive behaviors (e.g., hand hygiene, social distancing). Information credibility was also related to compliance with health recommendations. Lep et al. examined the relationship between information source perceived credibility and trust, and participants' levels of self-protective behavior among 1,718 Slovenians (age range: 18–81 years, 81.7% female). The authors found that scientists, general practitioners (family doctors), and the National Institute of Public Health were perceived as the more credible source of information, while social media and government officials received the lowest ratings. Perceived information credibility was found to be associated with lower levels of negative emotional responses (e.g., nervousness, helplessness) and a higher level of observance of self-protective measures (e.g., hand washing). Siebenhaar et al. also studied the link between compliance, distress by information, and information avoidance. They examined the online survey responses of 1,059 adults living in Germany (Mage = 39.53, 79.4% female). Their results suggested that distress by information could lead to higher compliance with preventive measures. Distress by information was also associated with higher information avoidance, which in turn is related to less compliance. Gantiva et al. studied the effectiveness of different messages regarding the intentions toward self-care behaviors, perceived efficacy to motivate self-care behaviors in others, perceived risk, and perceived message strength, in a sample of 319 Colombians (age range: 18–60 years, 69.9% female). Their experiment included the manipulation of message framing (gain vs. loss) and message content (economy vs. health). Participants judged gain-frame health related messages to be stronger and more effective in changing self-behavior, whereas loss-framed health messages resulted in increased perceived risk. Rahn et al. offer a comparative view of compliance and risk perception, examining three hazard types: COVID-19 pandemic, violent acts, and severe weather. With a sample of 403 Germans (age range: 18–89 years, 72% female), they studied how age, gender, previous hazard experience and different components of risk appraisal (perceived severity, anticipated negative emotions, anticipatory worry, and risk perception) were related to the intention to comply with behavioral recommendations. They found that higher age predicted compliance with health recommendations to prevent COVID-19, anticipatory worry predicted compliance with warning messages regarding violent acts, and women complied more often with severe weather recommendations than men.

A fourth line of research examined media use, mental health and well-being during the COVID-19 pandemic. Gabbiadini et al. addressed the use of digital technology (e.g., voice/video calls, online games, watching movies in party mode) to stay connected with others during lockdown. Participants, 465 Italians (age range: 18–73 years, 348 female), reported more perceived social support associated with the use of these digital technologies, which in turn was associated with fewer feelings of loneliness, boredom, anger, and higher sense of belongingness. Muñiz-Velázquez et al. compared the media habits of 249 Spanish adults (Mage = 42.06, 53.8% female) before and during confinement. They compared the type of media consumed (e.g., watching TV series, listening to radio, watching news) and found the increased consumption of TV and social networking sites during confinement to be negatively associated with reported level of happiness. People who reported higher levels of well-being also reported watching less TV and less use of social networking sites. Majeed et al., on the other hand, examined the relation between problematic social media use, fear of COVID-19, depression, and mindfulness. Their study, involving 267 Pakistani adults (90 female), suggested trait mindfulness had a buffer effect, reducing the impact of problematic media use and fear of COVID-19 on depression.

Taken together, these findings highlight how using different frames for mass media gives a more expansive view of its positive and negative roles, but also showcase the major concerns in the context of a pandemic crisis. As limitations we highlight the use of cross-sectional designs in most studies, not allowing to establish true inferences of causal relationships. The outcome of some studies may also be limited by the unbalanced number of female and male participants, by the non-probability sampling method used, and by the restricted time frame in which the research occurred. Nevertheless, we are confident that all the selected studies in our Research Topic bring important and enduring contributions to the understanding of how media, individual differences, and social factors intertwine to shape our lives, which can also be useful to guide public policies during these challenging times.

## Author Contributions

PA: conceptualization, writing the original draft, funding acquisition, writing—review, and editing. FE: conceptualization, writing—review, and editing. MP: writing—review and editing. NP: conceptualization, writing the original draft, writing—review, and editing. All authors approved the submitted version.

## Conflict of Interest

The authors declare that the research was conducted in the absence of any commercial or financial relationships that could be construed as a potential conflict of interest.

## Publisher's Note

All claims expressed in this article are solely those of the authors and do not necessarily represent those of their affiliated organizations, or those of the publisher, the editors and the reviewers. Any product that may be evaluated in this article, or claim that may be made by its manufacturer, is not guaranteed or endorsed by the publisher.

